# Child and Adolescent Psychiatry: Unmet Needs Before and After the COVID-19 Pandemic

**DOI:** 10.3390/children12020218

**Published:** 2025-02-12

**Authors:** Matteo Chiappedi

**Affiliations:** Child and Adolescent Neuropsychiatry Unit, ASST Pavia, 27029 Vigevano, Italy; matteo.chiappedi@unipv.it

The SARS-CoV-2 pandemic formally began on 11 March 2020, when the World Health Organization declared it as an evolution of the Public Health Emergency of International Concern (PHEIC). Only in May 2023 did the World Health Organization reduce the classification to an “established and ongoing health issue”. What happened in these 3 years had not been foreseen and provided undeniable evidence that modern day medicine still has limitations, but also holds great potential (with the COVID-19 vaccines being the symbol of the latter).

A number of changes in everyday life were necessary; in many cases, these changes were also established by law. Many of them could be seen as potential negative factors for children and adolescents’ psychological wellbeing. One could divide these factors into three main groups—direct consequences of the pandemic (e.g., having parents or friends ill or fearing to be infected, difficulties in receiving medical and psychological support, etc.); changings in protective everyday experiences (e.g., avoiding friends, sports and leisure activities of all kinds and replacing them with solitary activities due to “stay-at-home” orders; school closures with distance learning often not readily and sufficiently applied; etc.); blame posed on “normal” requests (e.g., in Italy, the need to protect fragile and older people led to blame being assigned to children and adolescents who tried to live their lives to the best of their ability within existing limitations).

Among the 17 contributions submitted to this Special Issue, 11 were accepted for publication (64.7%). These papers can be divided into two main groups according to their topic.

The first group investigated more general aspects of child and adolescent psychiatry, which was highly relevant both before and after the SARS-Cov-2 pandemic. The possibility of coping with adolescents’ risky behaviors was studied by Natali et al. [1]. They provided evidence that a single workshop combining psychoeducation and skills training, which lasted for only 3 h, could improve teachers’ confidence and feelings of potential self-efficacy to respond to these behaviors. The authors evidenced the potential of online sessions to increase the efficacy of this intervention and to offer it to a larger number of teachers.

Cossu et al. [2] studied 55 adolescents presenting with suicidal ideation and/or suicide attempt. They used the Minnesota Multiphasic Personality Inventory—Adolescents, evidencing statistically significant differences between the two groups. Adolescents who attempted suicide had higher levels of relational difficulties, more risky behaviors, reduced aspirations for their future, and also a higher tendency to lie, as well as an increased use of repression. These data, although needing to be confirmed in larger prospective studies, offered a possible way to support strategies to stratify suicide attempt risk and, therefore, interventions.

This leads to the second group of papers, i.e., those studying what happened to children and adolescents during the SARS-CoV-2 pandemic. Pontillo et al. [3] compared the number of inpatients followed in their Child and Adolescent Neuropsychiatry Unit before and during the pandemic in Italy. They showed a significant reduction in the number of inpatients during the peak of the pandemic and an increase in the following months, supporting the idea that “stay at home” orders had also reduced access to treatments. This is even more relevant because the authors also showed that mood disorders, non-suicidal self-injurious behaviors and suicidal ideation increased significantly during the “stay at home” period (also named “lockdown”).

This increase in mood symptoms was confirmed by Shakarun et al. [4] in their study conducted in Saskatchewan, Canada. Their study involved 563 child–parent dyads and showed that hybrid learning, disrupted activities and increased screen time worsened mood alterations, as well as being part of an ethnic minority and living in smaller cities.

Pedrini et al. [5] studied in detail how changes in lifestyle habits due to the SARS-CoV-2 pandemic acted on anxiety in a group of adolescents. According to their regression analyses, increases in anxiety were associated with sleep problems, difficulties in reducing screen time and loneliness. Interestingly, their data seem to suggest a possible long-term effect of the pandemic and of the public health measures (including “stay at home” orders), both in changing lifestyle habits and their purpose and impact on one’s mental health.

Akin and Sarrar [6] conducted a cross-sectional case–control study in Germany. Their data confirmed the presence of more atypical aspects of personality development, coupled with a less mature defense style and higher levels of psychodynamic conflicts in adolescents with mental health issues, leading to a number of disorders involving the body (somatoform symptoms, eating disorders, and alcohol use disorders) and/or mood (depressive disorders) and/or anxiety. During the pandemic period, these adolescents showed lower levels of conflict on the topic of autonomy (i.e., taking care of oneself versus being cared for by parents in a more or less passive way).

The role of parenting was studied by Facci et al. [7] in Italy during the pandemic period. They enrolled 136 mothers of preschool children for a survey and used multiple regression analyses to show an association between warmth and negative feelings on the one hand and positive parenting on the other; this was moderated by dismissing parental style toward children’s emotions. These data support the importance of sharing emotions and feelings, although negative and difficult to think about, to improve childrens’ development, even in times of crisis.

Working on even younger subjects, Richter et al. [8] studied infant regulatory problems, comparing their incidence in the pandemic versus the post-pandemic period in Germany. Crying/whining/sleeping problems and excessive crying had a higher prevalence in the post-pandemic period and were associated with less positive parenting behaviors in both mothers and fathers, this being partially mediated by parenting stress. This proved the importance of addressing infant mental health and to see parenting stress as a possible entry point for therapeutic interventions.

School closure in the context of “stay at home” orders was another disruption of children’s and adolescents’ “life routine”. Berger et al. [9] studied the possibility for school mental health professionals in Australia to support and address the mental health needs of young people during the SARS-CoV-2 pandemic. These authors evidenced a number of relevant concerns regarding remote evaluations and counseling, both in terms of the reliability of assessment tools and of ethical issues.

These aspects are even more important in view of the findings of Lacombe et al. [10]. These authors interviewed 184 pre-adolescents using self-reported questionnaires. They found an increase in school burnout compared to what had been obtained in a similar survey in 2014. In this respect, the factors with the greatest impact were reduced confidence in the future, perceived stress, parental support and mathematics results; other significant factors were somatic symptoms and a negative and non-supportive classroom climate.

The Russo-Ukrainian war started in 2022 and provided an example of a non-health crisis, whose effects on adolescents were compared to those of the SARS-CoV-2 pandemic by Šalčiūnaitė-Nikonovė et al. [11]. These authors showed high levels of significant anxiety during both periods, although predictors were different (stress, problematic social media use and female gender for the pandemic period; stress, loneliness and lower self-efficacy for war period).

It is sad to have to face these kind of “natural experiments” [12]; as far as they are not avoidable by the best efforts of the scientific community, it is at least our duty to exploit them to improve our understanding and interventions.
